# The incidence and trends of laryngeal malignancies in Sri Lanka from 2001 to 2019: a Joinpoint analysis of the national cancer database

**DOI:** 10.1186/s43046-023-00163-6

**Published:** 2023-02-21

**Authors:** Umesh Jayarajah, Ravindri Jayasinghe, Ashan Fernando, Sanjeewa Seneviratne

**Affiliations:** Department of Surgery, Faculty of Medicine, University of Colombo, Kynsey Road, , P.O. Box 271, Colombo 8, Western Province Sri Lanka

**Keywords:** Age-standardised rate, Incidence, Laryngeal cancer, National cancer registry, Sri Lanka

## Abstract

**Introduction:**

Laryngeal cancer is the ninth commonest cancer among Asian males. Global and regional epidemiological analyses have shown varying patterns in the incidence and risk factors for laryngeal cancer. Therefore, we aimed to analyse the trends in the incidence and histological patterns of laryngeal cancers for the first time in Sri Lanka.

**Methods:**

We used the population-based Sri Lanka cancer registry data and pooled all newly diagnosed patients with laryngeal malignancies from 2001 to 2019 (a 19-year study period). The WHO age-standardised incidence rates (ASR) were calculated using the WHO standard pollution. We used the Joinpoint regression software to calculate the estimated annual percentage change (EAPC) and analysed the trends in the incidence by different age categories and sex.

**Results:**

From 2001 to 2019, 9808 new cases of laryngeal cancers (males = 8927, 91%, mean age = 62 years) were registered. The incidence of laryngeal cancers was greatest in the 70–74-year followed by 65–69-year age groups. Around 7.9% were reported as carcinoma not otherwise specified (NOS). Squamous cell carcinoma (90.1%) was the commonest documented histology type. A rise in the WHO-ASR was noted from 1.91 per 100,000 in 2001 [95% confidence interval (95% CI): 1.69–2.12] to 3.59 per 100,000 in 2017 [(95% CI: 3.34–3.84); EAPC: 4.4 (95% CI: 3.7–5.2), *p* < 0.05 for trend] followed by a decrease in the incidence [2.97 per 100,000 in 2019 (95% CI: 2.74–3.2), EAPC: − 7.2 (95% CI: − 21.1–9.1, *p* > 0.05)]. From 2001 to 2017, the proportional increase in incidence was greater in males than females [EAPC: 4.9 (95% CI: 4.1–5.7 vs. 3.7 (95% CI: 1.7–5.6)].

**Conclusions:**

We identified an increasing incidence of laryngeal cancer in Sri Lanka from 2001 to 2017 followed by a slight decrease. Further studies are essential to identify the aetiological factors. Development of laryngeal cancer prevention and screening programmes for high-risk populations may be considered.

## Introduction

Globally, laryngeal cancer is predominantly seen among men. Laryngeal cancers are estimated to account for 0.25% of all cancers among males and 0.13% of all cancer-related mortality [1]. The global occurrence of laryngeal cancers among women is relatively rare [1]. The estimated male to female ratio (7:1) is one of the greatest discrepancies in cancer by gender [1]. The estimated annual incidence of laryngeal cancer in 2017 was 2.76 cases per 100,000 population, with a mortality rate of 1.66 deaths per 100,000 population [2]. The regions of high incidence were the Caribbean and Central and Eastern Europe, Southern Europe and Western Asia [1]. The differences in the prevalence of risk factors were postulated as the main reason for wide regional variations in the incidence and survival of laryngeal cancers [3]. Furthermore, laryngeal cancers were attributed to a considerable burden of health loss averaging around 3.28 million disability-adjusted life year per annum [2].

Laryngeal cancer incidence has been shown to be rising among Asian men, and the highest increase in the age-standardised incidence rate (ASR) was noted in East Asia [1, 4]. In neighbouring India, the reported incidence of laryngeal cancer was between 1.26 and 8.18 per 100,000 population, in different regions of the country [3] with a wide variation in the incidence being observed among different populations in India. Furthermore, laryngeal cancer was responsible for 17,560 deaths in India in the same year [3]. According to the national cancer database in Sri Lanka in 2019, the estimated ASR based on the standard world population for males and females were 5.6 and 0.4 per 100,000 respectively [5].

The National Cancer Control Programme (NCCP) is the primary national institution in Sri Lanka responsible for cancer data gathering, including the maintenance of the Sri Lanka Cancer Registry (SLCR). Since 1985, nationwide coverage of cancer data was implemented and at present covers all government and private institutions including hospitals and pathology laboratories dealing with cancer care [6]. As per the regional variations observed in Asia and neighbouring India, analysing the trends of laryngeal cancers in Sri Lanka has become a timely necessity for the implementation of future national policies. As a result, we set out to investigate the trends in the incidence of laryngeal cancer in Sri Lanka. Moreover, the age-related and sex-related variations in the laryngeal cancer incidence rates have also been analysed.

## Methods

All newly diagnosed laryngeal malignancies were identified from the annually published SLCR data from 1 January 2001 to 31 December 2019 [5]. Laryngeal cancers were classified and documented according to the International Classification of Diseases-10 (ICD-10) system (CD32). The age, sex and histology types of laryngeal cancers were collected from the published data. For each year, age-standardised incidence rates (ASR) of laryngeal malignancies were calculated by sex per 100,000 population, based on the WHO age-standardised populations [7]. The incidence rates were calculated for different age groups such as 0–44, 45–59, 60–74 and 75 + years and sex. Previously, similar approaches were used to analyse the patterns of other common malignancies in Sri Lanka [8, 9].

The Joinpoint regression analysis software can help you identify spots in a linear incidence curve where a statistically significant change has happened over time [10]. We used the Joinpoint regression analysis software (version 4.3) to calculate the estimated annual percentage change (EAPC) and to analyse the trends in the incidence by different age categories and sex. The detailed methodology has been described in previous publications [8, 9, 11]. Using a Monte Carlo permutation approach, the smallest number of Joinpoints of statistical significance were identified [12]. A *p* value of less than 0.05 was considered significant.

## Results

**Fig. 1:**
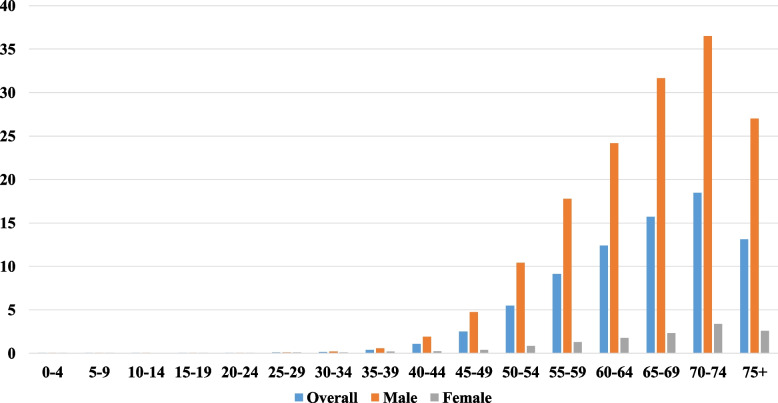
Laryngeal cancer incidence with age from 2001 to 2019 (per 100,000 population) in Sri Lanka

From 2001 to 2019, a total of 9808 newly diagnosed laryngeal cancers were retrieved (males = 8927, 91%, male to female = 10.1). The patients’ mean age was 62 years (males 62.1, females 61.3 years). The incidence of laryngeal cancers was greatest in the 70–74-year followed by 65–69-year age groups (Fig. 1).

The details on histology were available for 16 years (a total of 7939 patients). Approximately 7.9% were recorded as carcinoma NOS (not otherwise specified). The majority (*n* = 7151, 90.1%) were squamous cell carcinoma (SCC). Other histological types included undifferentiated carcinoma (*n* = 30, 0.4%), adenocarcinoma (*n* = 29, 0.4%) and small cell carcinoma (*n* = 18, 0.2%).

Details about the anatomical classification of laryngeal cancers were available for 16 years (a total of 7939 patients). The commonest region was the glottis (49.1%, *n* = 3898) followed by the supraglottic region (29.2%, *n* = 2312). Other regions were laryngeal cartilage (2.36%, *n* = 187) and subglottic region (1.26%, *n* = 100), and the rest were classifying as larynx NOS (17.8%, *n* = 1412).

**Fig. 2:**
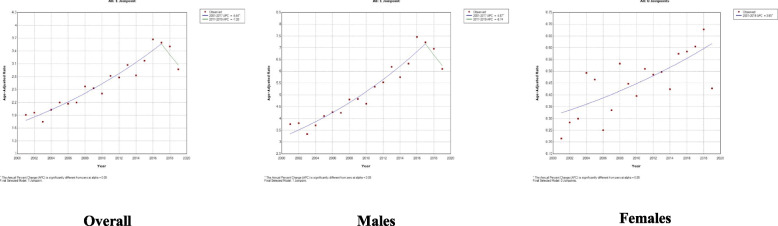
Joinpoint regression analysis of WHO standardised incidence rates in laryngeal cancer from 2001 to 2019 including all histology types in Sri Lanka

The Joinpoint analysis of trends revealed that the WHO-ASR of laryngeal malignancies in Sri Lanka has increased from 1.91 per 100,000 in 2001 [95% confidence interval (95% CI): 1.69–2.12] to 3.59 per 100,000 in 2017 (95% CI: 3.34–3.84); [EAPC: 4.4 (95% CI: 3.7–5.2), *p* < 0.05 for trend] followed by a decrease in the incidence [2.97 per 100,000 in 2019 (95% CI: 2.74–3.20), EAPC: − 7.2 (95% CI: − 21.1–9.1), *p* > 0.05]. A greater proportional rise in incidence was noted in males than females (Fig. 2).

**Fig. 3:**
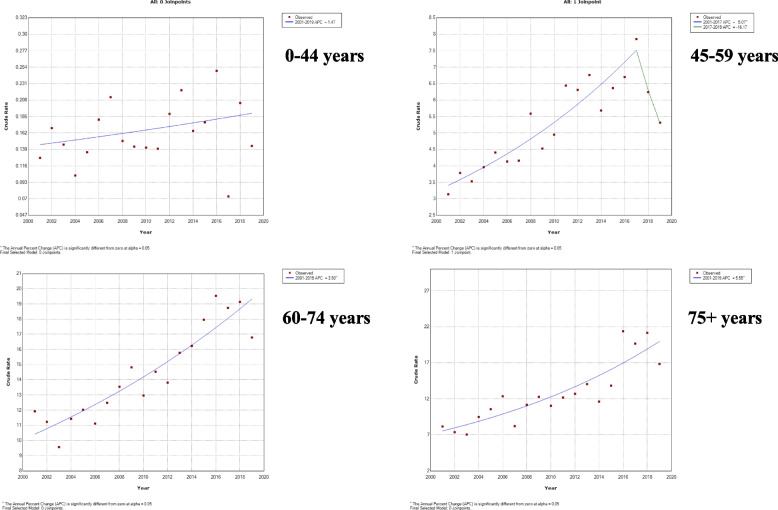
Joinpoint regression analysis of age-categorised trends in the incidence of laryngeal cancer including all patients from 2001 to 2019 in Sri Lanka

**Fig. 4:**
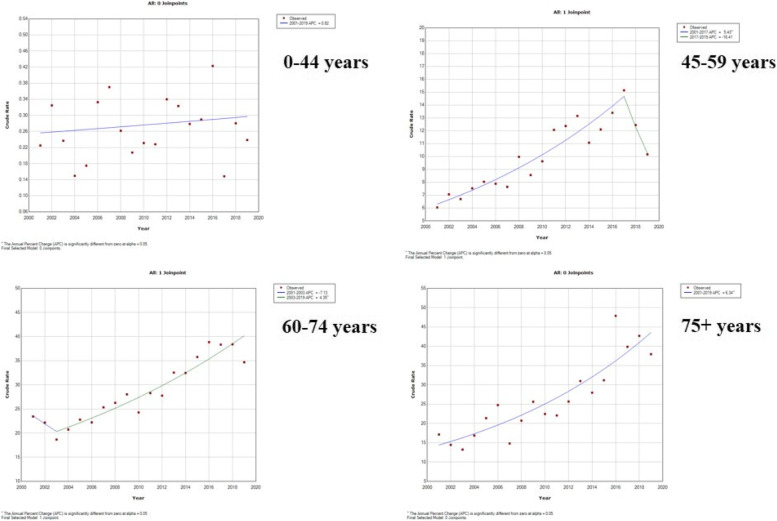
Joinpoint regression analysis of age-categorised trends in the incidence of laryngeal cancer among males from 2001 to 2019 in Sri Lanka

**Fig. 5:**
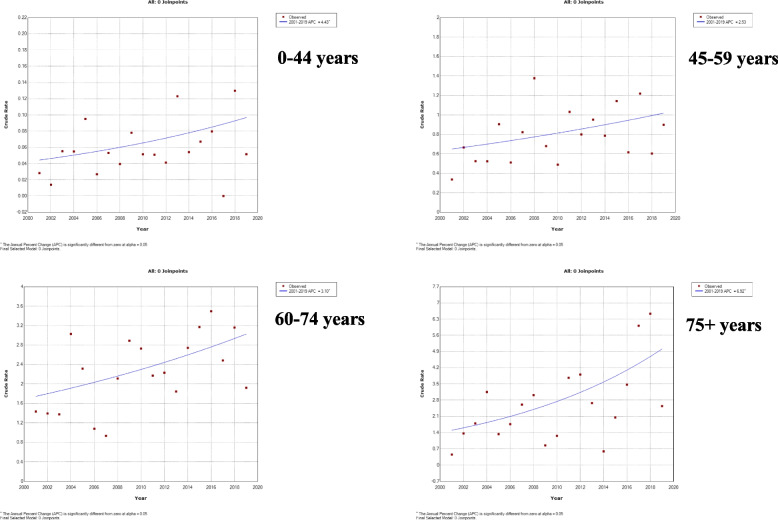
Joinpoint regression analysis of age-categorised trends in the incidence of laryngeal cancer among females from 2001 to 2019 in Sri Lanka

The total population was analysed in relation to the age-related incidence of laryngeal cancer. Varying trends of incidence were noted among different age categories. The 0–44-year age group showed a growing trend in incidence, with an EAPC of 1.5 (95% CI: − 0.8–3.8, *p* > 0.05). From 2001 to 2017, the 45–59-year age group showed a growing trend with an EAPC of 5.1 (95% CI: 3.8–6.3, *p* 0.05), followed by a non-significant falling trend. Both 60–74-year and 75 + year age groups showed significantly increasing trends. The 75 + age group had the greatest increase in incidence [EAPC: 5.6 (95% CI: 4.1–7.0)] (Fig. 3). A similar trend was also noted among males where a general rising incidence was seen among all age categories with the highest increase in the 75 + age group [EAPC of 4.8 (95% CI: 7.9–8.9, *p* < 0.05] (Fig. 4). Females aged 0–44 [EAPC: 4.4 (95% CI: 0.3–8.7)], 45–59 [EAPC: 3.1 (95% CI: 0.4–5.9)], and 75 + [EAPC: 6.9 (95% CI: 2.4–11.6) showed statistically significant rising trends (Fig. 5).

## Discussion

The global incidence of laryngeal malignancies is rising, along with an increase in incidence and mortality especially in the South Asian region [13, 14]. Similar to the world trends and that of the developing countries in the region, the present study that utilised the nationwide cancer data from the SLCR showed a predominant increase in the trend of laryngeal cancer in the country up to 2017 followed by a slight decrease. This increasing trend was probably the combined effect of an actual increase together with advancements in cancer diagnosis and reporting.

Sri Lanka is a country with a free public healthcare system with a substantial contribution from private health systems. The high cost of cancer care in the private sector overrides the affordability by the majority of the Sri Lankan population. As a result, the public health system bears more than 90% of the burden of cancer care in Sri Lanka [15]. Therefore, studying the incidence patterns of all cancers including laryngeal cancer will help the government allocate resources and make important policy decisions to allocate funds to accommodate the rise.

The observed increase is likely to have been contributed by improvements in coverage in national cancer data collection and improvements in diagnosis. The coverage of the NCCP had increased in Sri Lanka over time and the number of data sources for the NCCP had also increased gradually over the years. However, an increase in the incidence was also noted when the national cancer registry’s data sources were consistent (i.e. from 2004 to 2005 and from 2008 to 2009). This supports the argument that a rise in the number of cancer diagnoses is a likely contribution to the rising incidence, probably related to the gradual advancement of healthcare facilities in both the public and the private health systems in the country. Despite all these possibilities, an apparent rise in incidence, comparable to that found in many neighbouring developing nations, suggests that a real increase in incidence is likely [14].

Several known risk factors are potential contributors towards this rising trend which include smoking, tobacco use, betel chewing and alcohol use [16]. However, globally, in addition to alcohol intake, betel chewing and tobacco, there are other risk factors including human papilloma virus (HPV) infection and occupational exposure in industries including woodwork and metal work and leather workers who are exposed to carcinogenic chemicals, dust and fumes as well as immunodeficiency states including post-transplantation [17].

In Sri Lanka, similar to the regional and global figures, the major risk factors appear to be tobacco smoking, betel chewing and alcohol due to their high prevalence. Katulanda et al. in 2010 showed that in Sri Lanka, the prevalence of smoking among men was 38%, which is much higher than in neighbouring nations [18]. Nevertheless, the prevalence of smoking among men has substantially decreased compared to 1994 where the prevalence was 54% [18]. Notably, female smoking prevalence was among the lowest in the world (0.6%), which probably was the main reason for the very low incidence of female laryngeal cancers [18]. According to the WHO, the prevalence of heavy episodic drinking is 31.7% among the adult population in Sri Lanka, which is seen almost exclusively among males [19]. However, the prevalence of other risk factors such as HPV is low in Sri Lanka. The prevalence of HPV infection in otherwise healthy women was 3.3% and the prevalence of oncogenic HPV genotypes such as 16 and 18 was 1.2% [20]. Furthermore, the national immunisation programme included HPV vaccination of girls in the recent decade. This will likely reduce the impact of HPV on laryngeal cancer in years to come.

In the present study, the overall incidence of laryngeal carcinoma according to age group and sex showed varying patterns. Because overall registry improvements are assumed to have affected all ages evenly, this may indicate a likely genuine rise in incidence. Moreover, the significant difference in the incidence of laryngeal carcinoma in males and females corresponds with the country’s different prevalence rates for alcohol and tobacco consumption among males and females. Therefore, it is important to implement strategies to control major risk factors including consumption of alcohol and tobacco smoking especially among high-risk age groups.

There are several limitations in this study. Firstly, there is an improvement in the coverage of data collection with regard to laryngeal carcinoma which might have contributed to the reported increase of laryngeal cancers. Improvements in diagnostics and accessibility of cancer care throughout time in our lower middle-income setting, as well as improved reporting, may have contributed to the reported rise in incidence. Although the study had showed a general increase in incidence across the adult population, certain age groups showed varying patterns. These differential changes cannot be explained purely due to increased coverage and suggest a component of a true increase in the incidence patterns. Analysis of risk factors, staging and mortality data may be helpful to overcome this limitation. However, the unavailability of data on risk factors, mortality, treatment setting, etc., in our lower middle-income setting precluded these analyses. Additional analysis of the stage at diagnosis and mortality would be helpful to evaluate the effectiveness of screening programmes for laryngeal cancers. The collection of risk factor information may also aid in better understanding the aetiology of the reported rise in laryngeal cancer incidence.

## Conclusions

Based on data from the National Cancer Registry, this study found an overall increase in the incidence of laryngeal cancer in Sri Lanka from 2001 to 2019, with men being much more affected. The increasing burden of laryngeal malignancies warrants further analyses to identify the proportional contribution of known causative agents towards this increase which will help target at-risk populations for both preventive strategies and screening programmes for early identification. Furthermore, a greater allocation of resources is necessary to deal with the rising burden of laryngeal carcinoma in Sri Lanka.

## Data Availability

Data used in this analysis is available from the corresponding author on reasonable request.

## Abbreviations

NCCP: National Cancer Control Programme
WHO: World Health Organization
ASR: Age-standardised incidence rates
EAPC: Estimated annual percentage change
CI: Confidence interval
NOS: Not otherwise specified
HPV: Human papilloma virus
